# Ultraviolet metasurface-enabled flat-top beam shaping with size preservation uniformity and broadband robustness

**DOI:** 10.1038/s41598-026-45434-z

**Published:** 2026-04-02

**Authors:** Wanting Li, Jie Li, Tianxiang Zhao, Yang Li, Chenxi Wang, Penggang Li, Ziwei Zheng, Fei Ding, Hongliang Li

**Affiliations:** 1 School of Integrated Circuits, Jiangnan University, Wuxi, 214122 China; 2 Key Laboratory of Multifunctional Nanomaterials and Smart Systems, Suzhou Institute of Nano-Tech and Nano-Bionics, Chinese Academy of Sciences, Suzhou, 215123 China; 3 State Key Laboratory of Materials for Integrated Circuits, Shanghai Institute of Microsystem and Information Technology, Chinese Academy of Sciences, Shanghai, 200050 China; 4 Future Display Institute, Xiamen University, Xiamen, 361000 China; 5 Digital Industry Research Institute, Zhejiang Wanli University, Ningbo, 315100 China; 6 School of Electronic Science and Technology, Eastern Institute of Technology, Ningbo, 315200 China

**Keywords:** Flat-top beam shaping, Metasurface, Size preservation, Ultraviolet, Engineering, Materials science, Optics and photonics, Physics

## Abstract

Ultraviolet lithography based on Gaussian beam sources plays a critical role in integrated circuit fabrication, yet the intrinsic strong central intensity and rapid edge decay of Gaussian beams often lead to blurred feature edges, tapered sidewalls, and pattern distortion during exposure. Converting Gaussian beams into flat-top beams while preserving the original beam size is therefore highly desirable but remains challenging for compact optical systems. In this work, we investigate metasurface-based Gaussian-to-flat-top beam shaping using two independent phase-design strategies: the geometrical mapping technique (GMT) and the iterative Fourier transform algorithm (IFTA). The GMT provides a deterministic reference solution based on energy redistribution, while the IFTA offers a flexible iterative optimization framework for controlling the output intensity distribution. Based on these design approaches, a hafnium dioxide nanopillar metasurface operating at a wavelength of 300 nm is proposed to generate flat-top beams with excellent beam-size preservation. Numerical simulations show that the generated flat-top beam exhibits an intensity uniformity of approximately 0.965 and a beam-shaping efficiency of about 79.5%, while the relative deviation between the flat-top beam width and the incident Gaussian beam width is maintained at only 0.24%. The metasurface also demonstrates stable beam-shaping performance across multiple target planes, over a broadband ultraviolet range from 240 to 360 nm, and under incident angles up to 10°. The proposed metasurface beam-shaping strategy provides a compact and versatile approach for generating uniform flat-top beams with strict beam-size control, offering promising potential for applications in ultraviolet lithography and laser micromachining.

**Supplementary Information:**

The online version contains supplementary material available at 10.1038/s41598-026-45434-z.

## Introduction

As the demand for smaller feature sizes and higher performance in integrated circuits grows, lithography, as the core process defining critical dimensions, faces increasingly stringent precision requirements^1^. Among various lithographic techniques, ultraviolet (UV) lithography has become a mainstream choice due to its high resolution and cost-effectiveness^2^. Deep ultraviolet lithography typically employs KrF (248 nm) or ArF (193 nm) lasers operating in the fundamental Gaussian mode. While Gaussian beams have been widely used for full-field and scanning exposures^3^, their high-peak central intensity and exponential edge decay often cause blurred pattern boundaries, tapered sidewalls, and exposure non-uniformity, necessitating multiple overlapping scans and frequent focus adjustments^4^. Moreover, stringent constraints on lithographic system architecture and process windows require precise control over beam characteristics. In this context, preserving the beam size, namely ensuring that the width of the output flat-top beam matches the diameter of the incident Gaussian beam, is essential for achieving high energy utilization and uniform exposure.

The critical impact of illumination quality on lithographic performance has been substantiated by recent studies. Gong et al.^5^ and Xue et al.^6^ demonstrated that illumination non-uniformity directly affects resolution, critical dimension control, and pattern fidelity. Similar requirements exist in other high-precision optical systems, such as micro-electromechanical systems light detection and ranging and micro-light-emitting diode displays^7,8^, where mismatches between beam size and system resolution lead to pixel crosstalk, optical loss, and degraded imaging performance. Comparable sensitivity to illumination uniformity and beam stability has also been reported in meta-lens-based imaging and measurement systems, such as digital image correlation, where non-uniform or unstable illumination can directly compromise measurement accuracy and system reliability^9^. In ultraviolet lithography, deviations between the illumination footprint and the designed exposure field have likewise been reported to induce critical dimension variations and reduced process margins, underscoring beam-size preservation as a system-level constraint rather than a secondary optimization target^10^. Despite its importance, beam-size preservation is rarely treated as a primary design constraint in existing UV beam-shaping approaches. Most reported solutions focus primarily on intensity redistribution, frequently resulting in beam expansion or shrinkage that necessitates bulky relay optics, thereby compromising lithographic fidelity and system integration.

To address non-uniform illumination, a variety of conventional beam-shaping techniques have been explored. Refractive optical systems enable intensity redistribution but rely on bulky components that limit system miniaturization and introduce additional optical loss^11^. Diffractive optical elements (DOEs) offer pixel-level phase control through computer-generated holography or iterative Fourier transform algorithms (IFTA), enabling flexible generation of complex light fields such as vortex beams, Bessel beams, and annular profiles^12–14^. However, in high-precision applications such as lithography, DOEs suffer from several fundamental limitations. A prominent issue is zero-order diffraction, where unmodulated energy concentrates along the optical axis, forming a high-intensity central hotspot that severely degrades flat-top uniformity and risks localized overexposure^15^. In addition, DOEs are inherently dispersive, with diffraction angles governed by $\:sin\theta\:\:\propto\:\:\lambda\: / d$, leading to narrow operational bandwidths and strong sensitivity to incident angles. Off-axis illumination modifies the effective optical path length, resulting in efficiency loss and the emergence of ghost diffraction orders^16^. Spatial light modulators provide programmability but are constrained by limited spatial resolution, which introduces interference artifacts and imperfect flat-top profiles^17^. Collectively, these limitations indicate that conventional beam-shaping techniques lack the capability to simultaneously control intensity uniformity, beam size, and robustness within a compact footprint.

Metasurface-based planar optics have emerged as a promising platform for compact and precise beam manipulation. Composed of subwavelength artificial meta-atoms, metasurfaces enable flexible control over phase, amplitude, and polarization by engineering localized electromagnetic responses^18–23^. A wide range of beam profiles, including flat-top beams^24^, Bessel beams^25^, and vortex beams^26^, have been demonstrated from microwave to near-infrared frequencies^27–29^. Nevertheless, extending these capabilities to the UV regime remains highly challenging due to strong material absorption and stringent fabrication tolerances, which severely constrain efficiency and scalability. Although phase retrieval methods are theoretically scalable to arbitrary input and output beam sizes, achieving beam-size preservation simultaneously with broadband operation and angular robustness remains an unresolved challenge in practical UV metasurface implementations.

**Fig. 1:**
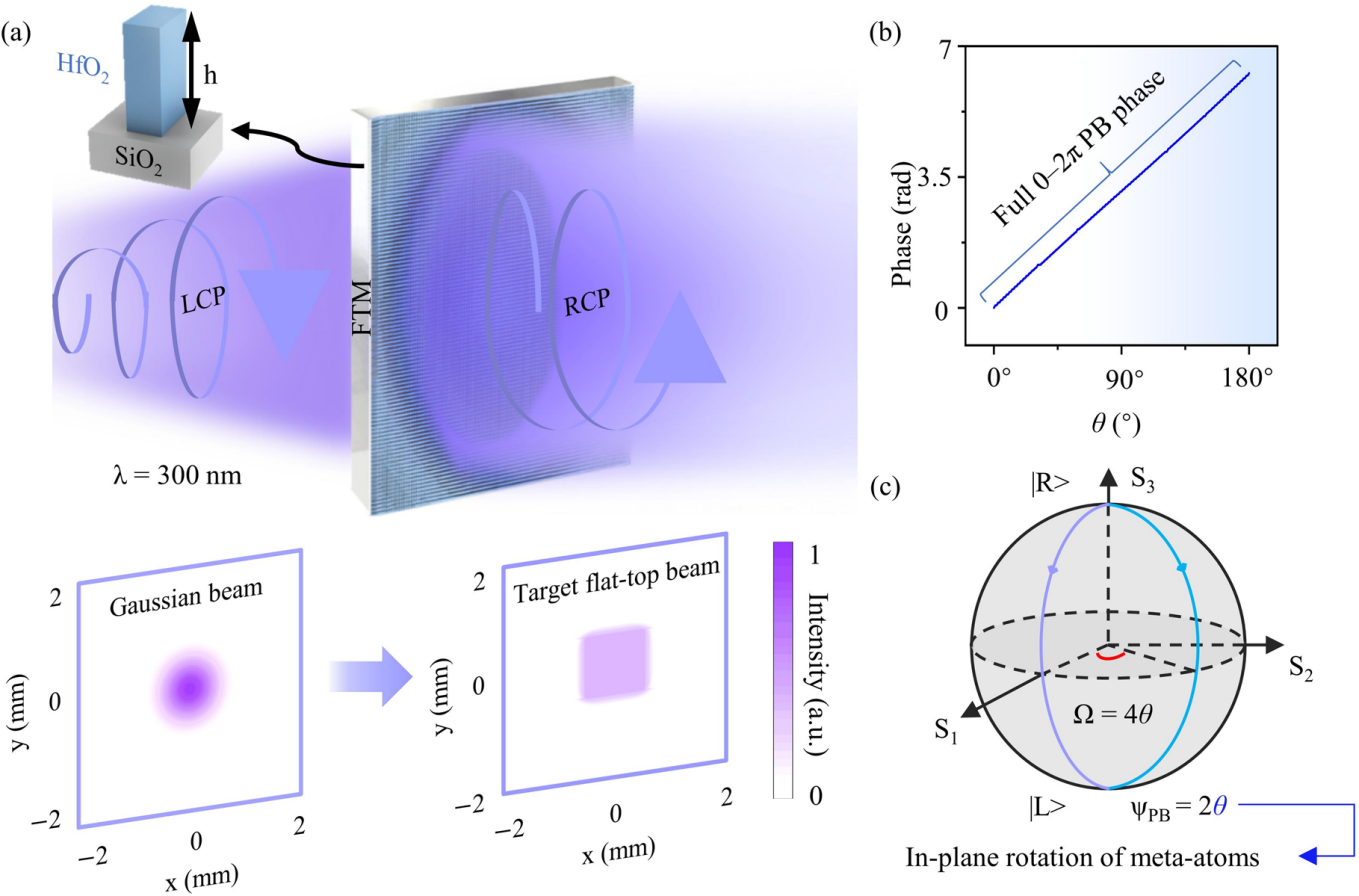
Gaussian to flat-top beam shaping mechanism of the flat-top metasurface (FTM). (**a**) An incident left circularly polarized (LCP) Gaussian beam at *λ* = 300 nm is transformed by the FTM into a highly uniform and highly efficient right circularly polarized (RCP) flat-top beam with preserved beam size. The schematic illustrates the incident Gaussian and output flat-top beams, and the inset depicts the structural design of the hafnium dioxide (HfO_2_) meta-atoms composing the FTM. (**b**) Simulated PB phase response under transmission conditions, demonstrating full 0–2π phase coverage as the orientation angle *θ* varies from 0° to 180°. (**c**) The polarization conversion process from LCP to RCP is illustrated on the Poincaré sphere.

A careful examination of existing literature reveals three fundamental bottlenecks that hinder the realization of robust UV flat-top metasurfaces. First, a trade-off exists between bandwidth and angular robustness. Dispersion-engineered structures designed for broadband performance often degrade rapidly under off-axis illumination due to effective optical path variations^30^. As a result, devices optimized for broadband operation typically exhibit limited angular tolerance, whereas wide-angle designs suffer from severe chromatic aberrations^31^. Second, and more critically for lithographic applications, a fundamental trade-off exists between intensity uniformity and beam-size preservation. Most inverse design and phase retrieval algorithms prioritize intensity mapping at a specific target plane while neglecting wavefront evolution. This omission leads to divergent or convergent beam propagation beyond the target plane, resulting in unstable beam dimensions and degraded lithographic precision^32^. Similar system-level challenges have been recognized in metasurface-based three-dimensional (3D) optical devices, where stable control of the optical field along both axial and transverse directions is essential for practical deployment^33^. Third, these geometric constraints are further exacerbated by material limitations in the UV regime. Unlike visible and infrared wavelengths, where high-index and low-loss materials such as silicon or titanium dioxide are readily available, the deep UV region suffers from strong absorption and a scarcity of wide-bandgap materials^34^. This severely restricts available aspect ratios and design freedom, making it increasingly difficult to satisfy phase, bandwidth, and robustness requirements simultaneously.

In beam-shaping problems, the phase distribution required to transform an incident beam into a prescribed target profile can be obtained using different design strategies. One commonly adopted approach is the geometrical mapping method (GMT), in which the relationship between the input and target intensity distributions is established through cumulative energy redistribution. Optical power is continuously reallocated from the Gaussian core to the flat-top plateau under the condition of energy conservation, enabling a deterministic coordinate mapping to be derived for calculating the required phase profile. Mapping-based beam-shaping techniques have been widely investigated in geometrical transformation optics and diffractive phase-element design, particularly for Gaussian-to-flat-top beam conversion^35–37^. Because of its deterministic formulation and early development in beam-shaping research, GMT provides a physically intuitive reference approach for Gaussian-to-flat-top beam transformation. Another widely used class of approaches relies on iterative phase-retrieval algorithms. Among them, the Gerchberg-Saxton (GS) algorithm represents one of the earliest iterative schemes for phase retrieval and wavefront reconstruction^38^. Building upon this class of iterative constraint methods, IFTA have been widely adopted for beam shaping and DOE design^39^. Compared with deterministic mapping strategies such as GMT, IFTA-based optimization operates on the global spatial field distribution and therefore provides greater flexibility in controlling far-field intensity patterns and incorporating multiple design constraints. These characteristics make IFTA particularly suitable for optical beam shaping problems where precise control of the output intensity distribution is required^40^.

In our work, the GMT and the IFTA-based phase-retrieval approach are independently employed for the design of Gaussian-to-flat-top beam-shaping metasurfaces. The GMT method is first introduced as a deterministic reference solution to examine the feasibility of size-preserved Gaussian-to-flat-top transformation. Subsequently, the IFTA-based strategy is investigated as an iterative optimization approach that allows more flexible control of the output intensity distribution. Corresponding phase distributions are generated using both design strategies, and metasurface structures are constructed on the basis of the obtained phase profiles. A HfO_2_ nanopillar FTM is designed and numerically investigated under ultraviolet illumination at *λ* = 300 nm. The flat-top beams generated by the FTM are evaluated at multiple target planes, across wavelengths ranging from 240 to 360 nm, and under incident angles from 0° to 10°. Comparative analysis of the simulation results shows that the two design strategies produce broadly comparable flat-top beam characteristics, while the IFTA-designed metasurface exhibits a slight advantage in selected metrics such as beam-size preservation. The presented strategy establishes a compact, versatile, and efficient approach for flat-top beam generation in the ultraviolet regime, addressing the critical requirement of beam-size preservation and providing potential applications in optical lithography and laser micromachining.

## Methods

### PB-phase-based metasurface realization

The beam shaping principle of the FTM is based on the Pancharatnam-Berry (PB) phase, realized through HfO_2_ nanopillars patterned on a silicon dioxide substrate. An incident LCP Gaussian beam at *λ* = 300 nm is converted into a RCP flat-top beam with high intensity uniformity and efficiency while maintaining beam size, as shown in Fig. 1a. As a wide-bandgap dielectric, HfO_2_ possesses a high refractive index ($\:{\it \mathrm{n}}_{{\mathrm{HfO}}_{\mathrm{2}}}\mathrm{\:>\:2.1}$) and a negligible extinction coefficient ($\:{\it \mathrm{k}}_{{{\mathrm{HfO}}_{{\mathrm{2}}} }} \approx {\mathrm{0}}$), providing an efficient and low-loss platform for UV phase modulation^41^. Transmission simulations demonstrate complete 0–2π PB phase coverage as the nanopillar orientation angle *θ* varies from 0° to 180°, as illustrated in Fig. 1b. As *θ* increases, the imparted PB phase varies linearly with the rotation angle. Therefore, 180 discrete orientation angles are selected, whose corresponding phases uniformly cover the full 0–2π range.

The polarization conversion and phase modulation of each birefringent meta-atom are governed by the Jones matrix:

1 $$\:\begin{array}{c}T\left(\theta\:\right)=R\left(\theta\:\right)\left[\begin{array}{cc}{e}^{i{\varphi\:}_{x}^{{\prime\:}}}&\:0\\\:0&\:{e}^{i{\varphi\:}_{y}^{{\prime\:}}}\end{array}\right]R\left(-\theta\:\right)\end{array}$$

where $\:{\mathrm{R(}}\theta {\mathrm{)}}$ represents the rotation matrix and $\:{\varphi\:}_{x}^{{\prime\:}}$, $\:{\varphi\:}_{y}^{{\prime\:}}$ denote the phase retardations. The incident and transmitted fields are related through $\:{E}_{out}=T\left(\theta\:\right){E}_{in}$^42^. Under circularly polarized illumination, the handedness of light is reversed from LCP to RCP, while acquiring a PB phase shift of 2*θ*. On the Poincaré sphere, as shown in Fig. 1c, this process corresponds to a trajectory from the north pole (LCP) to the south pole (RCP), where the accumulated geometric phase equals half of the subtended solid angle, $\:{\phi\:}_{PB}=\varOmega\:/2=2\theta\:$, revealing its geometric origin. Continuous phase modulation required for realizing the beam-shaping phase profile derived in the subsequent sections is enabled by PB-phase modulation.

### Energy-conserving Gaussian-to-flat-top formulation

The incident Gaussian beam exhibits an excessively strong central intensity and exponentially decaying edges, described by:

2 $$\:\begin{array}{c}{I}_{GB}\left(x,y\right)={I}_{0}\mathrm{exp}\left(-\frac{{2x}^{2}}{{\omega\:}_{0}^{2}}-\frac{{2y}^{2}}{{\omega\:}_{0}^{2}}\right)\end{array}$$

where $\:{I}_{0}$ is the peak intensity and $\:{\omega\:}_{0}$ denotes the $\:1/{e}^{2}$ beam radius. Following transformation by the FTM, the output beam forms a uniform square flat-top profile of width 2R × 2R, ideally expressed as:

3 $$\:\begin{array}{*{20}c} {I_{{FB}} = \left\{ {\begin{array}{*{20}l} {I_{f} ,} \hfill & {\left| x \right| \le \:R,\left| y \right| \le \:R} \hfill \\ {0,} \hfill & {otherwise} \hfill \\ \end{array} } \right.} \\ \end{array}$$

Energy conservation requires the total power $\:{P}_{GB}$ of the Gaussian beam:

4 $$\:\begin{array}{c}{P}_{GB}=\iint\:{I}_{0}\mathrm{exp}\left(-\frac{{2x}^{2}}{{\omega\:}_{0}^{2}}-\frac{{2y}^{2}}{{\omega\:}_{0}^{2}}\right)dxdy={I}_{0}\frac{\pi\:{\omega\:}_{0}^{2}}{2}\end{array}$$

to equal that of the flat-top beam:

5 $$\:\begin{array}{c}{P}_{FB}=\iint\:{I}_{f}dxdy={I}_{f}{\left(2R\right)}^{2}=4{R}^{2}\cdot\:{I}_{f}\end{array}$$

yielding $\:{I}_{f}=\pi\:{\omega\:}_{0}^{2}/8{R}^{2}\cdot\:{I}_{0}$. Instead of defining beam-size preservation using the $\:{\omega\:}_{0}$, the size-preserved condition in this work is established by equating the flat-top side length to the full width at half maximum (FWHM) of the incident Gaussian beam, namely $\:2R={W}_{FWHM}$. Here, the Gaussian FWHM is related to the beam radius by $\:{W}_{FWHM}={\omega\:}_{0}\sqrt{2ln2}$. The corresponding flat-top plateau amplitude satisfies:

6 $$\:\begin{array}{c}\frac{\left|{E}_{f}\right|}{\left|{E}_{0}\right|}=\sqrt{\frac{{I}_{f}}{{I}_{0}}}=\sqrt{\frac{\pi\:}{4ln2}}\approx\:1.06\end{array}$$

The slight increase in plateau amplitude originates from spatial redistribution of optical energy rather than any violation of energy conservation.

**Fig. 2:**
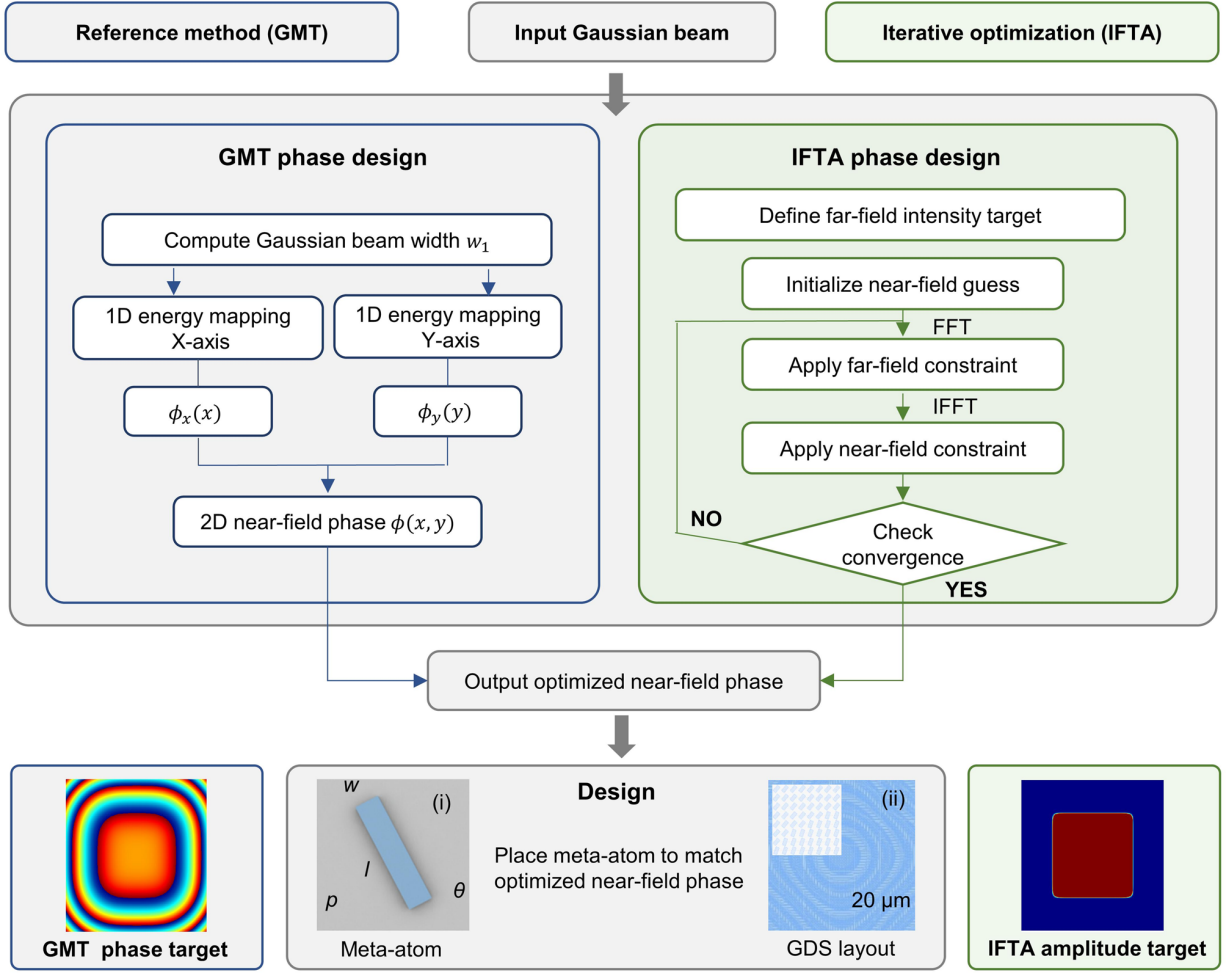
Workflow of the metasurface phase design using GMT and IFTA. Starting from the incident Gaussian beam, the GMT approach analytically constructs a near-field phase profile by establishing a size-preserving energy mapping between the Gaussian input and the desired flat-top distribution. In contrast, the IFTA approach determines the phase distribution through iterative optimization, where the optical field is repeatedly propagated between the metasurface plane and the target plane with amplitude constraints enforced in both planes. The resulting phase distribution is then translated into nanopillar orientation angles for metasurface realization. (i) Schematic of the HfO_2_ nanopillar meta-atom, with parameters including period* p*, length* l*, width *w*, height ℎ, and in-plane rotation angle *θ*. (ii) Local GDS layout of the metasurface designed using an IFTA-based phase design workflow, with a size of 150 × 150 µm^2^. The inset shows an enlarged local view of the graphic data system (GDS) layout, highlighting the arrangement of the meta-atoms, with a scale bar of 20 μm.

### Physics-informed phase optimization

To implement the energy-conserving and size-preserving Gaussian-to-flat-top beam transformation in a realizable metasurface, the phase distribution is designed using two approaches. One approach analytically constructs the phase profile based on an energy-redistribution principle GMT derived from Gaussian-to-flat-top mapping, while the other determines the phase distribution through iterative optimization using the IFTA implemented in the Meta-Component module of PlanOpSim. In the analytical construction, a physically consistent target distribution is formulated using an energy-redistribution strategy based on geometric transformation. A Gaussian beam incident along the optical axis with a waist diameter $\:{\omega\:}_{1}$ located at z = 0 is considered, where the metasurface is positioned to reshape the beam into a flat-top profile of diameter $\:{\omega\:}_{2}$ at a propagation distance *d*. Under a one-to-one cumulative energy mapping between the incident Gaussian beam and the desired flat-top profile^35,36,43^, the phase distribution along the x direction is derived as:

7 $$\:\begin{array}{c}\varphi\:\left(x\right)=\frac{2\pi\:}{\lambda\:d}{\int\:}_{-D/2}^{x}\left[{\omega\:}_{2}\left(\frac{S\left({x}^{{\prime\:}}\right)}{S\left(D/2\right)}-\frac{1}{2}\right)-{x}^{{\prime\:}}\right]d{x}^{{\prime\:}};\left|x\right|<\frac{D}{2}\end{array}$$

where *λ* is the incident wavelength and *D* denotes the lateral extent of the computational window. The function $\:S\left(x{\prime\:}\right)={\int\:}_{-D/2}^{x'}exp(-8{\xi\:}^{2}/{\omega\:}_{1}^{2})d\xi\:$ represents the cumulative energy distribution of the incident Gaussian beam within $\:[-D/2,x']$. Normalization of $\:S\left(x^{\prime}\right)/S(D/2)$ ensures accurate correspondence between the Gaussian input and the flat-top output distributions. The orthogonal 1D phase solutions are subsequently combined to form a two-dimensional (2D) phase distribution $\:{\varphi\:}_{GMT}(x,y)$.

In the IFTA-based phase design, the phase distribution is obtained through iterative optimization under prescribed amplitude constraints. The IFTA belongs to the GS algorithm family^44,45^ and is widely used for phase retrieval problems. In this framework, the complex optical field can be written as $\:U\left(x,y\right)=A(x,y){e}^{i{\varphi\:}_{IFTA}(x,y)}$ where $\:A(x,y)$ and $\:{\varphi\:}_{IFTA}(x,y)$ enote the amplitude and phase distributions, respectively. During the optimization process, the amplitudes at the metasurface plane and the target plane are specified according to the incident Gaussian beam and the desired flat-top intensity distribution, while the phase distribution is iteratively updated to satisfy these constraints.

The optical field is repeatedly propagated between the metasurface plane and the target plane using Fourier and inverse Fourier transforms. The iterative update can be expressed in the general form as:

8 $$\:\begin{array}{c}{U}_{k+1}={\varsigma\:}_{spatial}\left({F}^{-1}\left[{\varsigma\:}_{freq}\left(F\left({U}_{k}\right)\right)\right]\right)\end{array}$$

where $\:F$ and $\:{F}^{-1}$ denote Fourier and inverse Fourier transforms, $\:{\varsigma\:}_{spatial}$ and $\:{\varsigma\:}_{freq}$ represent the constraint operators at the metasurface and target planes, respectively. During each iteration, the field is forward propagated to the target plane, where the calculated amplitude is replaced by the desired distribution while preserving the propagated phase. The field is then inversely propagated back to the metasurface plane, where the amplitude is restored to the incident Gaussian amplitude, thereby enforcing the phase-only modulation condition of the metasurface. The procedure is repeated until the intensity error at the target plane falls below a predefined threshold. The iterative optimization yields an optimized phase distribution $\:{\varphi\:}_{IFTA}(x,y)$. The optimized phase distributions $\:{\varphi\:}_{GMT}(x,y)$ and $\:{\varphi\:}_{IFTA}\left(x,y\right)$ are subsequently implemented to design the corresponding metasurfaces.

## Results and discussion

### Phase implementation and structural design of the HfO_2_ metasurface

Based on the phase design strategies described above, a unified PB phase-based HfO_2_ metasurface platform was adopted to implement the target phase profiles derived from different design methods and to enable a fair comparison of their beam-shaping performance. Under circularly polarized illumination, continuous 0–2π phase modulation was obtained by rotating the anisotropic meta-atoms according to the in-plane angle *θ*^46^, as shown in Fig. 2i. The meta-atom geometry was optimized through parametric sweeps using the Meta Cell module of the PlanOpSim platform (Belgium), yielding dimensions of length *l* = 150 nm, width *w* = 50 nm, height *h* = 500 nm, and a unit cell period *p* = 320 nm. The planar GDS layout of the entire FTM is shown in Fig. 2ii, illustrating the spatial arrangement of the optimized meta-atoms.

### Quantitative metrics for flat-top beam evaluation

Consistent evaluation of size-preserved Gaussian-to-flat-top beam shaping and objective comparison between different phase-design strategies require a unified set of quantitative metrics. The shaping efficiency *η* is defined as the ratio of the optical power contained in the generated flat-top beam within its effective flat-top region to the total optical power of the incident Gaussian beam:

9 $$\:\begin{array}{c}\eta\:=\frac{{\iint\:}_{\left(x,y\right)\in\:\varOmega\:}{I}_{FB}(x,y)dxdy}{{\iint\:}_{all}{I}_{GB}(x,y)dxdy}\times\:100\%\end{array}$$

Here, $\:{E}_{FB}(x,y)$ and $\:{E}_{GB}(x,y)$ denote the complex electric field distributions of the generated flat-top beam and the incident Gaussian beam, respectively. The corresponding intensity distributions are defined as: $\:{I}_{FB}\left(x,y\right)={\left|{E}_{FB}(x,y)\right|}^{2}$, $\:{I}_{GB}\left(x,y\right)={\left|{E}_{GB}(x,y)\right|}^{2}$. The integration domain $\:\varOmega\:$ represents the effective flat-top region of the flat-top beam and is defined as$\:{\Omega\:}=\left\{\left.\left(x,y\right)\right|{I}_{FB}(x,y)\ge\:0.5{I}_{FB,max}\right\}$, where $\:{I}_{FB,max}$ is the maximum intensity of the flat-top beam. Identification of the effective flat-top region based on the above criterion provides a consistent description of the target beam area and enables quantitative evaluation of energy redistribution from the incident Gaussian beam into the flat-top region. Beam quality is further characterized by three complementary metrics describing plateau uniformity, edge transition characteristics, and beam-width stability. First, the intensity uniformity $\:U$ of the flat-top beam is evaluated based on the statistical variation of intensity within the central flat-top plateau. To exclude edge-transition effects and to focus on the intrinsic uniformity of the flat-top region, the uniformity is calculated over a restricted high-intensity region defined as $\:{{\Omega\:}}_{U}=\left\{\left.\left(x,y\right)\right|{I}_{FB}(x,y)\ge\:0.95{I}_{FB,max}\right\}$. The uniformity $\:U$ is then expressed in terms of the standard deviation * σ* of the intensity distribution within $\:{{\Omega\:}}_{U}\:$^47^:

10 $$\:\begin{array}{c}U=1-\frac{\sigma\:}{\stackrel{-}{I}}\end{array}$$

Here, $\:\stackrel{-}{I}$ denotes the average intensity of all sampling points within the region $\:{{\Omega\:}}_{U}$. The standard deviation σ is defined as:

11 $$\:\begin{array}{c}\sigma\:=\sqrt{\frac{1}{N}\sum\:_{i=1}^{N}{\left({I}_{i}-\mu\:\right)}^{2}},\:\stackrel{-}{I}=\frac{1}{N}{\sum\:}_{i=1}^{N}{I}_{i}\end{array}$$

where $\:N$ is the number of sampling points, $\:{I}_{i}$ is the intensity at each point. The standard deviation σ is used to quantify the intensity fluctuations within the flat-top region. Second, the edge steepness $\it \:\mathrm{k}$ is defined as the slope obtained from a linear fit to the intensity profile between the points corresponding to 10% and 90% of the peak intensity:

12 $$\:\begin{array}{c}k=\frac{n{\sum\:}_{i=1}^{n}\left({x}_{i}{I}_{i}\right)-{\sum\:}_{i=1}^{n}{x}_{i}{\sum\:}_{i=1}^{n}{I}_{i}}{n{\sum\:}_{i=1}^{n}{x}_{i}^{2}-{\left({\sum\:}_{i=1}^{n}{x}_{i}\right)}^{2}}\end{array}$$

where $\it \:\mathrm{n}$ indicates the number of fitting points, $\it \:{\mathrm{x}}_{\mathrm{i}}\:$is the spatial coordinate, and $\it \:{\mathrm{I}}_{\mathrm{i}}$ is the corresponding intensity value. To eliminate dependence on the absolute intensity scale, a normalized edge steepness $\:{k}_{norm}$ is further introduced:

13 $$\:\begin{array}{c}{k}_{norm}=\frac{k\varDelta\:{x}_{10-90}}{\stackrel{-}{I}\:}\end{array}$$

where $\:\varDelta\:{x}_{10-90}$ is the corresponding spatial interval between the 10% and 90% intensity points. Dimensionless normalization enables comparison of edge-transition characteristics under different intensity levels. Smaller values of $\:{k}_{norm}$ correspond to sharper edge transitions, whereas larger values indicate broader edges and stronger degradation of the flat-top profile. Finally, beam-width stability is quantified by the flat-top beam width $\:{W}_{FB}$, defined as the width at half of the average flat-top intensity, and compared with the Gaussian beam width $\:{W}_{GB}={W}_{FWHM}$, enabling assessment of the beam-size preservation capability of the FTM.

### Performance of the GMT-designed flat-top metasurface

**Fig. 3:**
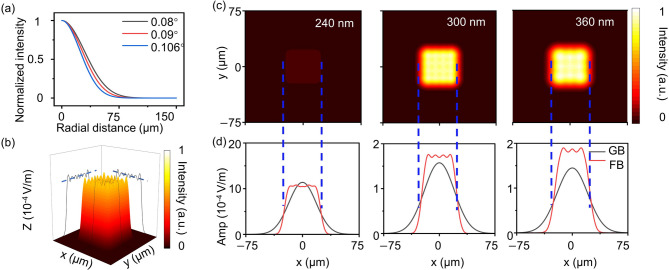
Beam shaping performance of the FTM designed using the GMT method for converting an incident Gaussian beam into a size-preserved flat-top beam. (**a**) Normalized radial intensity profiles of the incident Gaussian beams used in the simulations. The half-divergence angles are 0.08°, 0.09°, and 0.106°, corresponding to the incident Gaussian beams at wavelengths of 240 nm, 300 nm, and 360 nm, respectively. (**b**) 3D intensity distribution of the generated flat-top beam at *λ* = 300 nm, together with cross-sectional views in the x-z and y-z planes. The blue dashed line indicates the average intensity within the flat-top region. (**c**) Simulated 2D intensity distributions of the generated flat-top beams at *λ* = 240 nm, 300 nm, and 360 nm, demonstrating consistent beam-shaping performance across multiple wavelengths. (**d**) Cross-sectional intensity profiles along the x direction comparing the incident Gaussian beam (black curves) and the generated flat-top beam (red curves). The blue dashed lines indicate the extracted beam width used to evaluate beam-size preservation.

The GMT, representing a deterministic mapping-based beam-shaping strategy, was first employed as a reference approach for size-preserved Gaussian-to-flat-top beam transformation. Systematic numerical simulations were carried out to evaluate the beam-shaping performance of the FTM designed using GMT. The simulations were performed using the Meta Component module of the PlanOpSim platform. Evaluation of the GMT-derived phase distribution was therefore conducted within the unified HfO_2_ metasurface framework introduced in Sect.  Phase implementation and structural design of the HfO_2_ metasurface. The operating wavelength of the metasurface was centered at 300 nm, while additional simulations were conducted at 240 nm and 360 nm in order to examine wavelength-dependent beam-shaping behavior. The incident field was defined as a single Gaussian beam. Maintenance of comparable transverse beam sizes at different wavelengths required the adoption of slightly different half-divergence angles for the incident Gaussian beams. The normalized radial intensity profiles of the incident beams are shown in Fig. 3a. Half-divergence angles of 0.08°, 0.09°, and 0.106° correspond to wavelengths of 240 nm, 300 nm, and 360 nm, respectively. The normalized intensity profiles exhibit the characteristic Gaussian distribution with a pronounced central peak and smooth radial decay. The Gaussian beam defined under these conditions therefore serves as the reference input distribution for subsequent beam-shaping analysis. Under the above incident conditions, the GMT-designed metasurface redistributes optical energy and forms a flat-top beam with a nearly uniform intensity plateau.

At the design wavelength of *λ* = 300 nm, the reshaped beam exhibits a well-defined flat-top intensity distribution. As illustrated in Fig. 3b, the generated beam forms a stable plateau region accompanied by relatively sharp edge transitions in the transverse plane. 3D intensity visualization together with the corresponding x-z and y-z cross sections confirm the formation of a uniform flat-top region. The cross-sectional intensity profiles in Fig. 3d further demonstrate that the output beam maintains nearly constant intensity within the plateau region while preserving the overall beam width relative to the incident Gaussian beam. Beam-size preservation is quantitatively evaluated using the relative width deviation $\:\left|{W}_{FB}-{W}_{GB}\right|/{W}_{GB}$. For the design wavelength of 300 nm, $\:{W}_{GB}=47.71\:\ \mu\:m$ and the extracted$\:\:{W}_{FB}=47.57\:\ \mu\:m$. The corresponding relative width deviation is only 0.28%, indicating that the GMT-designed metasurface effectively maintains the transverse beam size during the Gaussian-to-flat-top transformation. In addition to beam-size preservation, several complementary metrics were used to characterize the beam quality. At 300 nm, the shaping efficiency reaches 79.5%, demonstrating efficient redistribution of optical energy into the target flat-top region. The intensity uniformity of the flat-top plateau is 0.976, indicating that the intensity variation within the central region remains relatively small. The edge steepness parameter is extracted as 1.21 × 10^− 4^ V/m, corresponding to a normalized edge steepness of 0.821, which reflects a well-defined transition between the flat-top plateau and the surrounding low-intensity region.

The beam-shaping behavior was further examined at wavelengths of 240 nm and 360 nm to evaluate the wavelength response of the GMT-designed metasurface. As shown in Fig. 3c, flat-top beam profiles can still be observed at both wavelengths, although the beam characteristics exhibit certain variations due to wavelength-dependent diffraction effects. At *λ* = 240 nm, $\:{W}_{GB}=42.56\:\ \mu\:m$ and the generated$\:{\:W}_{FB}=48.42\:\ \mu\:m$, corresponding to a relative width deviation of 13.76%. The intensity uniformity remains 0.976, and the normalized edge steepness is 0.822, indicating that a relatively uniform plateau can still be obtained, although the beam-size preservation deteriorates at this wavelength. The shaping efficiency decreases significantly to 6.1%, reflecting the increased diffraction mismatch between the designed phase profile and the shorter wavelength. At *λ* = 360 nm, $\:{W}_{GB}=48.58\:\ \mu\:m$ and the extracted $\:{W}_{FB}=47.57\:\ \mu\:m$, corresponding to a relative width deviation of 2.88%. The intensity uniformity is 0.974, the normalized edge steepness is 0.827, and the shaping efficiency reaches 79.4%. Such results indicate that the flat-top beam characteristics remain relatively stable for wavelengths longer than the design wavelength. Additional analyses of the wavelength-dependent propagation characteristics and the applicability of the GMT-based beam-shaping formulation to different wavelength regimes and material platforms are provided in Supplementary Sections S1 and S2. Overall, GMT-based phase design enables deterministic Gaussian-to-flat-top beam shaping while maintaining excellent beam-size preservation at the design wavelength. The obtained results therefore provide a reference solution for size-preserved flat-top beam generation and establish a baseline for comparison with iterative phase-design approaches discussed in the following section.

### Performance of the IFTA-designed flat-top metasurface

**Fig. 4:**
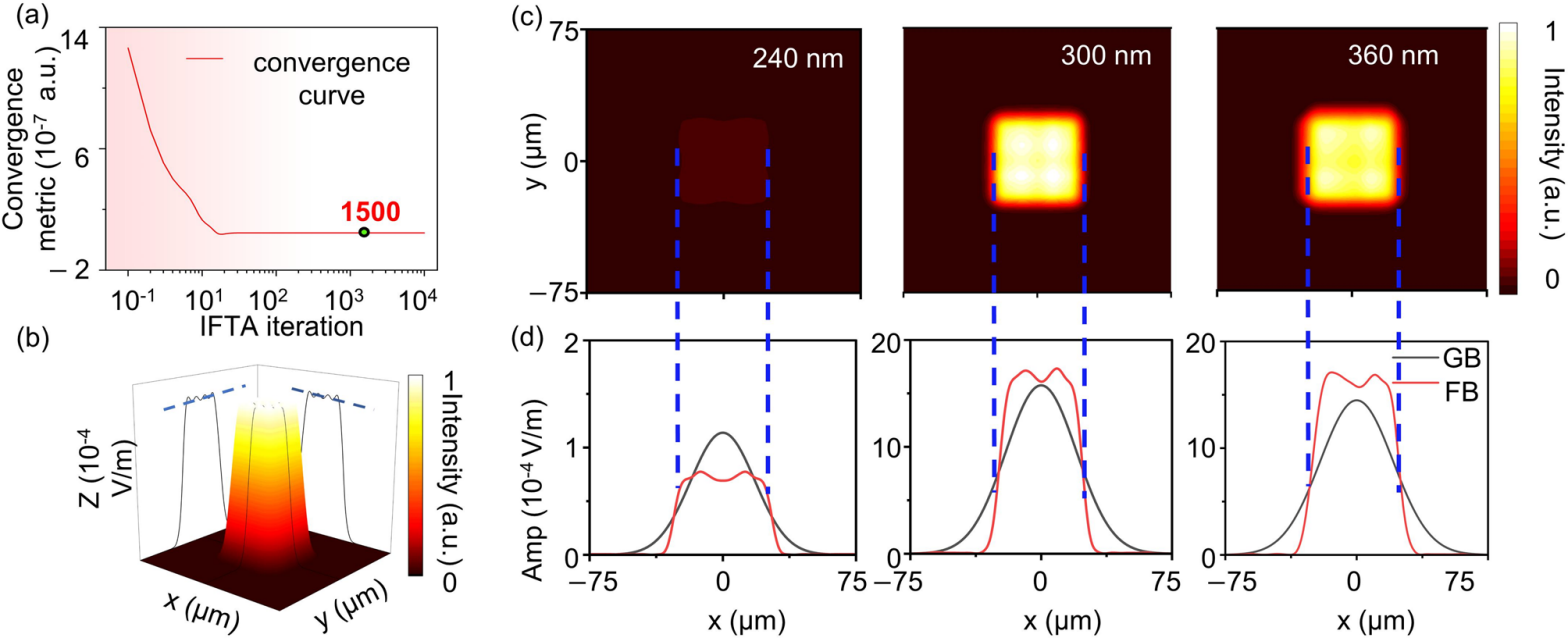
Beam-shaping performance of the FTM designed using the IFTA method for converting an incident Gaussian beam into a size-preserved flat-top beam. (**a**) Convergence curve of the IFTA optimization process. The convergence metric gradually decreases with increasing iteration number and reaches a stable value after approximately 1500 iterations, indicating that the iterative phase optimization has converged. (**b**) 3D intensity distribution of the generated flat-top beam at *λ* = 300 nm, together with cross-sectional views in the x-z and y-z planes. The blue dashed line indicates the average intensity within the flat-top region. (**c**) Simulated 2D intensity distributions of the generated flat-top beams at *λ* = 240 nm, 300 nm, and 360 nm, illustrating the beam-shaping performance of the metasurface at different wavelengths. (**d**) Cross-sectional intensity profiles along the x direction comparing the incident Gaussian beam (black curves) and the generated flat-top beam (red curves). The blue dashed lines indicate the extracted beam width used to evaluate beam-size preservation.

Following the deterministic GMT-based design presented in the previous section, the IFTA was further adopted as an alternative phase-design strategy for Gaussian-to-flat-top beam shaping. In contrast to mapping-based approaches, IFTA belongs to a class of iterative phase-retrieval and Fourier-domain optimization algorithms that provide flexible control of the target intensity distribution. Such characteristics make IFTA particularly suitable for beam-shaping and DOE design tasks involving prescribed far-field intensity profiles. Numerical simulations were performed using the Meta Component module of the PlanOpSim platform in order to evaluate the beam-shaping capability of the metasurface designed with IFTA. The same metasurface platform introduced in Sect.  Phase implementation and structural design of the HfO_2_ metasurface was employed, ensuring that performance differences originate primarily from the phase-design strategy rather than structural variations of the device. The metasurface was optimized for a design wavelength of 300 nm, while additional simulations were conducted at wavelengths of 240 nm and 360 nm for analysis of wavelength-dependent beam-shaping behavior. The angular response of the device was also examined for incident angles of 0°, 5°, and 10°. The convergence behavior of the IFTA optimization process was first investigated. The convergence metric decreases rapidly during the early stage of iteration and approaches a stable value after approximately 20 iterations, indicating that the phase distribution quickly approaches an optimal solution. In the present implementation, the iterative procedure was extended to 1500 iterations to ensure numerical stability of the final phase profile. As illustrated in Fig. 4a, the convergence metric remains nearly unchanged after the early convergence stage, confirming that the optimization process has reached a stable solution.

Under the optimized phase distribution, the metasurface reshapes the incident Gaussian beam into a flat-top beam characterized by a clearly defined intensity plateau. The 3D intensity distribution of the generated beam at 300 nm is shown in Fig. 4b, where a uniform central region is observed together with gradual intensity transitions toward the beam edges. Cross-sectional views along the propagation direction further verify the formation of a stable flat-top profile. The wavelength-dependent beam-shaping behavior is illustrated in Fig. 4c and d. At the design wavelength of 300 nm, the extracted beam widths are$\:{\:W}_{GB}=47.71\:\ \mu\:m$ and $\:{W}_{FB}=47.59\:\ \mu\:m$, corresponding to a relative width deviation of only 0.24%. Such a small deviation reflects excellent preservation of the transverse beam size during the Gaussian-to-flat-top transformation. The shaping efficiency reaches 79.5%, while the intensity uniformity and normalized edge steepness are 0.965 and 0.838, respectively. The obtained beam profile therefore combines efficient energy redistribution with high spatial stability of the plateau region.

**Fig. 5:**
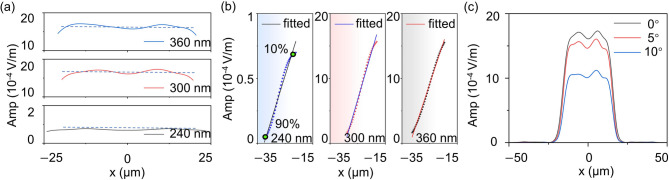
Wavelength response and angular tolerance of the IFTA-designed FTM. (**a**) Cross-sectional beam profiles of the generated flat-top beams along the x direction at wavelengths of 360 nm, 300 nm, and 240 nm. The blue dashed lines represent the average intensity within each flat-top region, illustrating the intensity uniformity of the generated flat-top beams at different wavelengths. (**b**) Edge-transition analysis of the flat-top beams, where the edge steepness is quantified by linear fitting between the 10% and 90% intensity levels (green markers) for wavelengths of 240 nm, 300 nm, and 360 nm. (**c**) Cross-sectional flat-top beam profiles at *λ* = 300 nm under different incident angles (0°, 5°, and 10°), demonstrating the angular tolerance of the designed metasurface.

At the shorter wavelength of 240 nm, beam-shaping performance becomes noticeably degraded. In this case,$\:{\:W}_{GB}=42.56\:\ \mu\:m$ and $\:{W}_{FB}=54.99\:\ \mu\:m$, corresponding to a width deviation of 29.2%. The shaping efficiency decreases to 5.8%, while the intensity uniformity and normalized edge steepness are 0.954 and 0.856, respectively. Although a flat-top-like distribution remains observable, energy concentration and beam-size preservation are strongly affected. Such degradation mainly arises from the increased phase mismatch between the designed phase profile and the shorter wavelength. The cross-sectional beam profiles at the three wavelengths are summarized in Fig. 5a, where the dashed lines represent the average intensity within the flat-top region. Several consistent trends can be identified. Beam-size preservation reaches the best performance at the design wavelength of 300 nm, remains relatively stable at 360 nm, and deteriorates significantly at 240 nm. The shaping efficiency follows a similar tendency, remaining close to 80% near the design wavelength while dropping sharply at shorter wavelengths. In contrast, the intensity uniformity varies only moderately across the examined wavelength range, indicating that once a flat-top plateau is formed, the central intensity distribution remains comparatively stable. The normalized edge steepness shows a slightly stronger wavelength dependence, suggesting gradual smoothing of edge transitions when the wavelength deviates from the design value, as illustrated by the edge-transition analysis in Fig. 5b.

The angular response of the metasurface was further examined at the design wavelength of 300 nm, as shown in Fig. 5c. As the incidence angle increases from 0° to 10°, the overall flat-top profile remains clearly recognizable, although the peak intensity gradually decreases. Such behavior originates primarily from geometric projection effects and reduced coupling efficiency under oblique illumination. Quantitatively, the shaping efficiency decreases from 79.5% at normal incidence to 69.4% at 5° and 47.7% at 10°. In contrast, the beam width remains relatively stable, with $\:{W}_{FB}=47.59\ \:\mu\:m$, 46.68 μm, and 46.72 μm for incidence angles of 0°, 5°, and 10°, respectively. Since$\:{\:W}_{GB}=47.71\ \:\mu\:m$, the corresponding width deviations are approximately 0.24%, 2.16%, and 2.08%. Meanwhile, the intensity uniformity remains close to 0.96 across the examined angles, and the normalized edge steepness varies only slightly from 0.838 to 0.861.

Taken together, the IFTA-designed metasurface enables efficient Gaussian-to-flat-top beam conversion while maintaining excellent beam-size preservation at the design wavelength. Stable plateau uniformity and well-defined edge transitions are maintained, and acceptable performance persists under moderate wavelength and angular variations. The results highlight the suitability of iterative phase-design strategies for beam-shaping tasks that require accurate control of far-field intensity distributions.

### Comparative analysis of flat-top beam shaping approaches

Two phase-design strategies, namely the Gaussian mapping technique GMT and the IFTA, were investigated in this work for the design of Gaussian-to-flat-top beam-shaping metasurfaces. Although both approaches aim to generate a uniform flat-top intensity profile, they originate from different theoretical frameworks and therefore exhibit distinct design characteristics. Mapping-based methods such as GMT belong to an earlier class of beam-shaping techniques. In such approaches, the required phase distribution is obtained through deterministic energy redistribution between the input and target intensity distributions under the constraint of energy conservation. By establishing a coordinate mapping between the Gaussian input beam and the desired flat-top output beam, the phase profile responsible for the beam transformation can be determined analytically. Owing to this deterministic formulation, GMT has been widely applied in geometrical beam transformation and wavefront engineering problems. Iterative algorithms such as IFTA follow a different design philosophy. Instead of explicitly constructing a coordinate mapping between the input and output beams, the target intensity distribution is gradually approached through repeated forward and backward propagation of the optical field between different planes while enforcing amplitude constraints. Because the optimization process occurs in the spatial-frequency domain and operates on the global field distribution, iterative algorithms provide greater flexibility for shaping complex far-field intensity profiles. Such characteristics explain the widespread use of IFTA-type algorithms in computer-generated holography and DOE design.

A direct comparison between the two design strategies obtained in this work is summarized in Table 1. Both GMT and IFTA achieve efficient Gaussian-to-flat-top conversion at the design wavelength of 300 nm. The shaping efficiency is identical for the two methods (79.5%), indicating comparable energy redistribution performance. Differences between the two solutions remain relatively small. The GMT-based design exhibits a slightly lower RMS intensity variation (2.4%) compared with the IFTA-based design (3.5%), while the IFTA-based design provides marginally better beam-size preservation with a width deviation of 0.24% compared with 0.28% for the GMT solution. These results suggest that both approaches are capable of achieving high-quality flat-top beam generation within the metasurface platform considered here.

Beyond the comparison between the two phase-design strategies used in this work, Table 1 also summarizes representative flat-top beam-shaping systems reported in the literature. Although operating wavelengths and optical configurations vary significantly among different studies, several general observations can be made by examining beam uniformity, shaping efficiency, and beam-size preservation. In spatial light modulation systems such asdigital micromirror device (DMD)- or spatial light modulator (SLM)-based configurations, flat-top beams are often generated through diffraction-order selection combined with spatial filtering. Extremely low root-mean-square intensity deviations below 0.3% have been reported in such systems. However, these implementations typically require additional optical components and relatively complex system configurations. DOE-based approaches combined with phase-retrieval algorithms such as the GS algorithm represent another widely used strategy. In such systems, flat-top beam formation results from diffraction-based redistribution of optical energy. For example, the system reported in . achieves an RMS intensity variation of approximately 2.87%, while metasurface designs combined with GS-type algorithms have demonstrated RMS deviations below 1.02%.

**Table 1:** Comparison of representative flat-top beam shaping approaches in terms of wavelength, propagation distance, efficiency, intensity uniformity, and beam-size deviation. (For consistency, the intensity uniformity of our work is re-evaluated using the RMS definition adopted in the corresponding references. For some prior works where beam-size variation was not explicitly reported, the corresponding values were extracted from published beam profiles and evaluated using the unified definition adopted in this work.)

	Approach	*λ*	*d*	*η*	RMS	$\:\frac{\left|{W}_{FB}-{W}_{GB}\right|}{{W}_{GB}}$
Ref.^48^	Dual-layer metasurface + GS	5 μm	130 μm	90%	< 1.02%	32%
Ref.^24^	Single metalens + extended GS	10.6 μm	5 × 10^4^ mm	93%	10%-12%	–
Ref.^49^	DOE + GS + spatial filtering	343 nm	10^5^ mm	–	2.87%	17.7%
Ref.^50^	DMD + LPF	633 nm	Image plane	30%-50%	< 0.3%	–
IFTA FTM	Metasurface + IFTA	300 nm	3590 μm	79.5%	3.5%	0.24%
GMT FTM	Metasurface + GMT	300 nm	2700 μm	79.5%	2.4%	0.28%

The metasurface-based implementations investigated in this work operate in a significantly different wavelength regime and rely on a compact single-layer optical platform. The IFTA-designed metasurface produces a flat-top beam with an RMS deviation of approximately 3.5% and a shaping efficiency of 79.5% at a wavelength of 300 nm and a propagation distance of 3590 μm. Although the RMS value is slightly higher than that reported in certain spatial filtering systems, the proposed design does not require auxiliary filtering components and therefore enables a considerably more compact optical implementation. A particularly notable feature emerges when beam-size preservation is considered. In many previously reported flat-top beam-shaping studies, changes in beam size are not treated as an independent design constraint. As indicated in Table 1, Gaussian-to-flat-top conversion in several approaches can introduce substantial beam expansion or compression. For instance, relative width deviations of approximately 32% in Ref.^48^ and 17.7% in Ref.^49^ indicate significant variations in the transverse beam size during the energy redistribution process.

In contrast, both metasurface designs investigated in this work maintain the transverse beam size of the generated flat-top beam very close to that of the incident Gaussian beam. Width deviations of 0.24% (IFTA) and 0.28% (GMT) indicate that the beam footprint is essentially preserved during the transformation process. Such capability is particularly relevant for applications in which the illumination area must remain consistent with the original beam geometry. For example, in ultraviolet lithography systems, variations in beam size may introduce nonuniform exposure or alignment errors within the optical system. Taken together, the results suggest that GMT and IFTA provide comparable beam-shaping performance within the metasurface framework considered here, with only minor differences in several performance metrics. The IFTA-based design offers slightly improved beam-size preservation, whereas the GMT-based solution yields marginally lower RMS intensity variation. More broadly, the analysis demonstrates that metasurface-based phase engineering provides an effective platform for achieving compact flat-top beam generation while maintaining precise control of beam dimensions.

## Conclusion

In summary, an IFTA-based beam-shaping design strategy was employed, while the GMT was also examined as a reference deterministic approach, to implement metasurfaces that reshape Gaussian beams into flat-top beams. At the design wavelength of 300 nm, the generated flat-top beams exhibit an intensity uniformity of approximately 0.965 and a beam-shaping efficiency of about 79.5%, while the relative deviation between the flat-top beam width and the incident Gaussian beam width is maintained at about 0.24%, indicating excellent beam-size preservation. The study is theoretical in nature, and all results are obtained under ideal numerical simulation conditions. Within the numerical simulation framework, systematic investigation of the wave-optical mechanisms governing the beam-shaping strategy and its intrinsic performance limits is enabled without the influence of experimental uncertainties. Based on designs optimized at discrete target planes, comprehensive numerical robustness analyses are performed to evaluate perturbation factors that are unavoidable in practical experiments, including axial misalignment around the designed planes, variations in incidence angle, broadband wavelength deviations, and changes in material platforms. In addition, the sensitivity of the GMT-based beam-shaping strategy to variations in the incident Gaussian beam divergence is analyzed in Supplementary Section S3. Stable flat-top beam profiles are maintained over a broadband ultraviolet spectral range centered at 300 nm with a bandwidth of ± 60 nm, covering wavelengths from 240 nm to 360 nm. In addition, high-quality flat-top beams are preserved under oblique incidence angles ranging from 0° to 10°. Robust beam-shaping performance is also demonstrated under variations in incident wavelength and metasurface material platforms. Within reasonable parameter ranges, beam-size preservation, high intensity uniformity, and high beam-shaping efficiency are consistently achieved. The robustness analyses provide numerical evidence supporting the feasibility of the design approach under realistic experimental tolerances.

Nevertheless, experimental realization remains a critical next step. The HfO_2_ nanopillars employed in the metasurface designs feature sub-100-nm lateral dimensions and relatively high aspect ratios, which are, in principle, compatible with state-of-the-art dielectric metasurface fabrication technologies such as electron-beam lithography. In practical fabrication processes, proximity effects, discretization of rotation angles, etching nonuniformity, and deviations in sidewall profiles may be introduced, leading to phase errors, polarization crosstalk, and efficiency degradation, particularly for large-area PB-phase-based metasurfaces. With the continued maturation of electron-beam lithography and related nanofabrication techniques, progressive mitigation of fabrication-induced challenges is expected through further process optimization. In conclusion, a metasurface design framework combining deterministic phase construction and iterative phase optimization is adopted for generating size-preserved flat-top beams with high intensity uniformity and high efficiency. Although the reported results are obtained from ideal numerical simulations, stable performance demonstrated under multiple perturbation conditions, together with the adoption of fabrication-compatible material platforms, indicates a clear pathway toward experimental implementation. Strong potential is therefore indicated for applications in ultraviolet lithography and other high-precision illumination systems.

## Supplementary Information

Below is the link to the electronic supplementary material.

Supplementary Material 1

## Data Availability

The datasets used and analyzed during the current study are available from the corresponding author upon reasonable request.
