# Supplementary material for: Ultraviolet metasurface-enabled flat-top beam shaping with size preservation uniformity and broadband robustness
Source: Sci Rep. 2026 Apr 2;16:15687. doi: 10.1038/s41598-026-45434-z (PMC13187159; doi:10.1038/s41598-026-45434-z)
Supplement: Supplementary file 1 — Supplementary Material 1 [file 41598_2026_45434_MOESM1_ESM.docx]

**Supplementary Information:**

**Ultraviolet metasurface-enabled flat-top beam shaping w****ith size preservation uniformity and broadband robustness**

Wanting Li^1^, Jie Li^1^, Tianxiang Zhao^2^, Yang Li^1^, Chenxi Wang^3,*^, Penggang Li^4^, Ziwei Zheng^5^, Fei Ding^6^, Hongliang Li^1,2,*^

^1^ School of Integrated Circuits, Jiangnan University, Wuxi 214122, China

^2^ Key Laboratory of Multifunctional Nanomaterials and Smart Systems, Suzhou Institute of Nano-Tech and Nano-Bionics, Chinese Academy of Sciences, Suzhou 215123, China

^3^ State Key Laboratory of Materials for Integrated Circuits, Shanghai Institute of Microsystem and Information Technology, Chinese Academy of Sciences, Shanghai 200050, China

^4^ Future Display Institute, Xiamen University, Xiamen 361000, China

^5^ Digital Industry Research Institute, Zhejiang Wanli University, Ningbo 315100, China

^6^ School of Electronic Science and Technology, Eastern Institute of Technology, Ningbo 315200, China

* Corresponding authors: Chenxi Wang ([wangcx77@mail.sim.ac.cn](mailto:wangcx77@mail.sim.ac.cn)) and Hongliang Li ([hlli2025@sinano.ac.cn](mailto:hlli2025@sinano.ac.cn)).

**S1. GMT-based broadband beam-shaping characteristics**


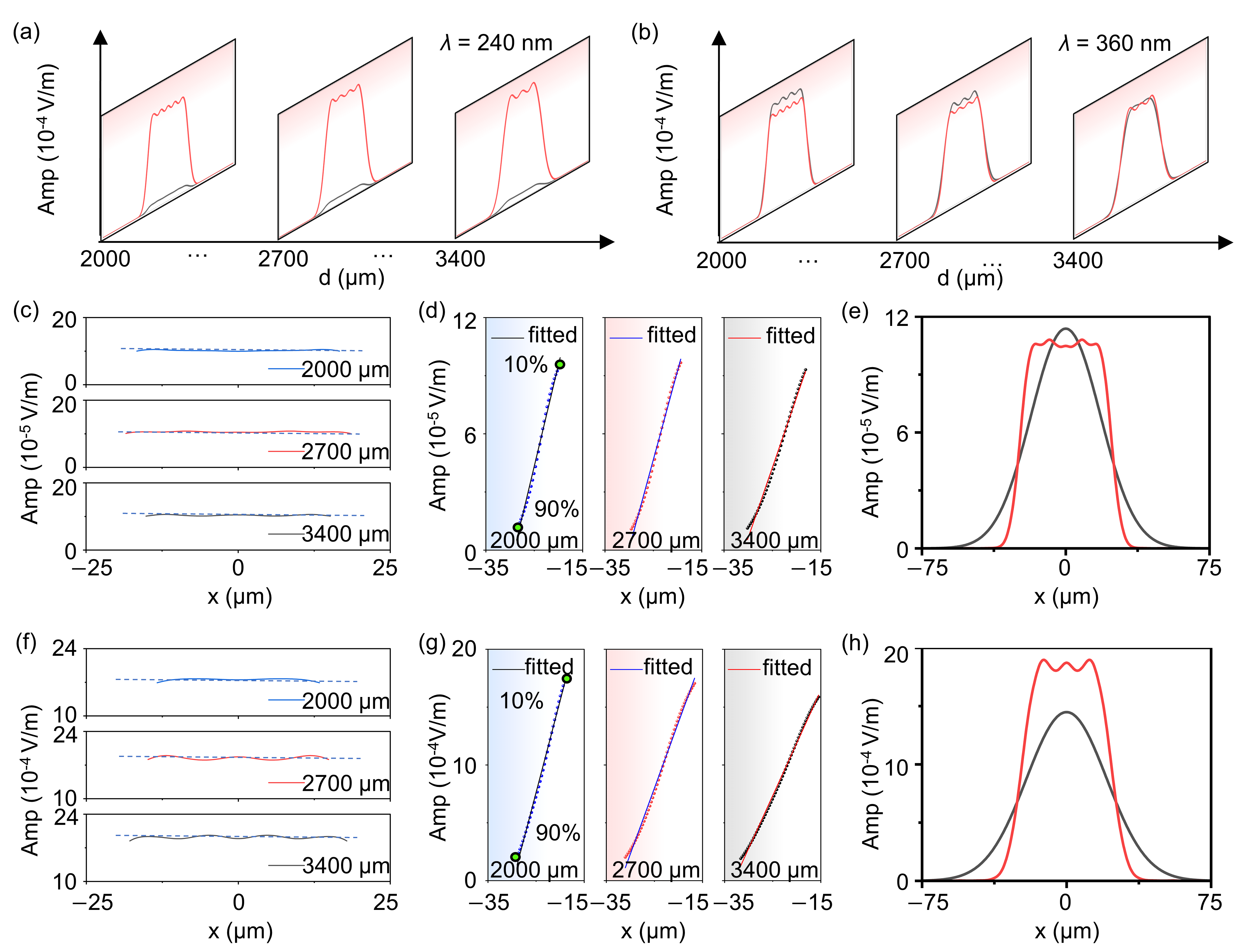


Fig. S1. The broadband robustness of the designed FTM for generating flat-top beams. (a) Flat-top beam at *λ* = 240 nm compared with that at *λ* = 300 nm under the same propagation distance, where the red line denotes the *λ* = 300 nm case. (b) Flat-top beam at *λ* = 360 nm compared with that at *λ* = 300 nm under the same propagation distance, where the red line denotes the *λ* = 300 nm case. At *λ* = 240 nm: (c) Beam profiles at propagation distances of 2000 μm, 2700 μm, and 3400 μm, with blue dashed lines representing the average intensity within each flat-top region. (d) The edge steepness of the flat-top beams in (c), quantified by the slope between the 10% and 90% intensity levels (green markers) using linear fitting. (e) Comparative cross-sectional intensity profiles of the incident Gaussian beam (black curves) and the modulated flat-top beam (red curves) at *d* = 2700 μm; At *λ* = 360 nm: (f) Cross-sectional profiles of the flat-top beams along the same propagation distances, with horizontal blue dashed lines marking the mean intensity of each flat-top region. (g) The slope of the flat-top beams in (f) is quantified in the same manner as in (d). (h) Comparative cross-sections at *d* = 2700 μm, illustrating the reshaped flat-top profiles against the original Gaussian input (black curves).

Additional simulations were performed to evaluate the wavelength adaptability of the Gaussian-to-flat-top beam transformation obtained using the GMT-derived phase profile. The beam-shaping performance was examined at ultraviolet wavelengths of 240 nm and 360 nm, while the design wavelength of 300 nm is included as a reference. The resulting beam profiles were evaluated at propagation distances of 2000 μm, 2700 μm, and 3400 μm under identical simulation conditions.

As shown in Fig. S1a and Fig. S1b, the incident Gaussian beam is reshaped into a flat-top intensity distribution at all examined wavelengths. The cross-sectional beam profiles confirm that the generated beams maintain a well-defined plateau region over the selected propagation distances. At *λ* = 240 nm, the extracted $W_{FB}$ are 48.14 μm, 48.37 μm, and 47.19 μm at propagation distances of 2000 μm, 2700 μm, and 3400 μm, respectively. The corresponding Gaussian reference width $W_{GB}$ is 42.56 μm, resulting in a relative width deviation of approximately 10%. The width deviation remains nearly unchanged across the examined propagation distances, indicating consistent beam-size characteristics under this wavelength condition. The plateau intensity distributions exhibit high spatial uniformity, as shown in Fig. S1c, with uniformity values of 0.985, 0.988, and 0.985. The normalized edge steepness values extracted from Fig. S1d are 0.8205, 0.8172, and 0.8218, indicating well-defined transitions between the flat-top plateau and the surrounding low-intensity region.

The corresponding results for *λ* = 360 nm are presented in Figs. S1f-h. The extracted flat-top beam widths $W_{FB}$ are 47.04 μm, 47.48 μm, and 46.38 μm, while the Gaussian reference width $W_{GB}$ is 48.93 μm, yielding a relative width deviation of approximately 5%. Compared with the shorter wavelength case, improved beam-size preservation is observed at this wavelength. The generated flat-top beams maintain clear plateau regions with uniformity values in the range of 0.983-0.988. The normalized edge steepness values are 0.8169, 0.8214, and 0.8077, indicating stable edge-transition characteristics. The persistence of flat-top beam profiles across different wavelengths is mainly associated with the non-resonant PB-phase modulation mechanism. Because PB phase arises from geometric rotation of anisotropic meta-atoms rather than resonance-based phase accumulation, the phase modulation exhibits relatively weak wavelength selectivity. Consequently, stable flat-top beam characteristics are preserved across multiple ultraviolet wavelengths.

**S2. Extension of the GMT-based beam-shaping analysis**


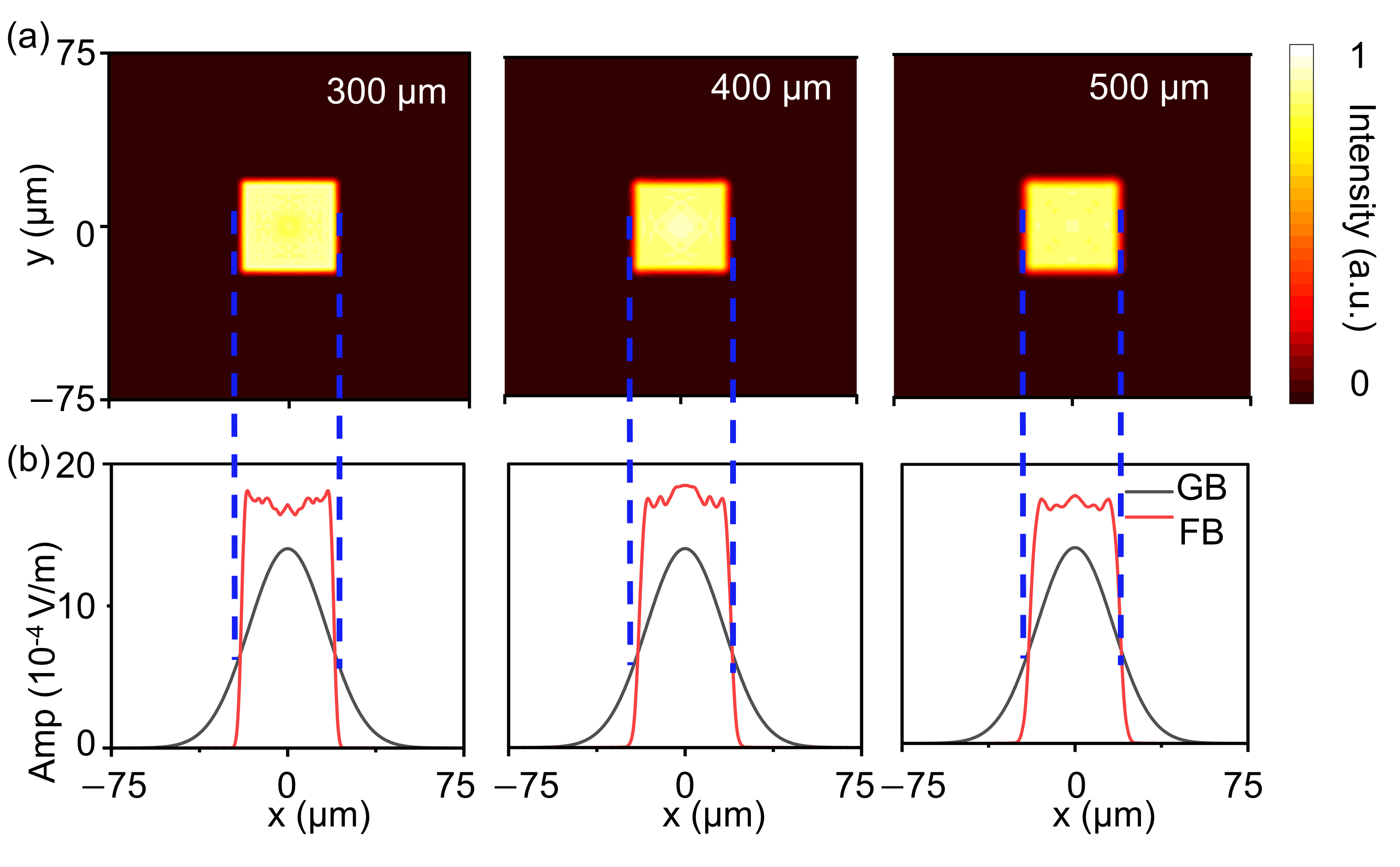


Fig. S2. (a) 2D intensity profiles of flat-top beams generated by the FTM at propagation distances of 300 μm, 400 μm, and 500 μm. (b) Comparative cross-sectional intensity distributions of the incident Gaussian beam (black curves) and the target flat-top beam (red curves) at these distances, demonstrating the effectiveness of the beam shaping.

An additional numerical investigation was conducted as an extension of the GMT-based beam-shaping analysis presented in the main text. The purpose of this supplementary study is to examine the behavior of the GMT-derived phase profile under a different wavelength regime and material platform. The metasurface design was extended to the visible spectral region with an operating wavelength of 532 nm. A polymer-based material platform was adopted using the photoresist IP-L, allowing evaluation under a substantially different refractive-index environment and fabrication-compatible material system. Compared with the hafnium dioxide metasurfaces operating in the ultraviolet regime, the IP-L platform represents a lower-index polymer material that is compatible with three-dimensional micro- and nano-printing techniques. The phase distribution used in the simulations follows the GMT-derived beam-shaping formulation, enabling examination of the beam characteristics under these modified physical conditions.

Under identical incident conditions, three independent metasurfaces were designed for target propagation distances of 300 μm, 400 μm, and 500 μm, respectively. Each metasurface was implemented using a phase profile corresponding to its designated target plane. The simulated two-dimensional intensity distributions and the corresponding one-dimensional cross-sectional profiles are shown in Fig. S2a and Fig. S2b. At the operating wavelength of 532 nm, the incident Gaussian beam is reshaped into a flat-top intensity distribution at the corresponding target planes. The generated beams exhibit clearly defined plateau regions together with distinct edge transitions. Quantitative analysis shows that the extracted flat-top beam widths are 39.59 μm, 39.08 μm, and 39.15 μm at propagation distances of 300 μm, 400 μm, and 500 μm, respectively. The corresponding Gaussian reference width is 38.71 μm, resulting in relative width deviations below 3%. These results indicate that the transverse beam size remains well preserved across the independently designed polymer metasurfaces. High spatial uniformity is maintained within the flat-top plateau region for all three designs, with uniformity values of 0.974, 0.970, and 0.983 at the respective target planes. The normalized edge steepness values are 0.8405, 0.8368, and 0.8279, indicating stable edge-transition characteristics of the generated flat-top beams.

The formation of stable flat-top beam profiles under these conditions indicates that the GMT-derived phase distribution retains its beam-shaping capability even when implemented on a different material platform and wavelength range. The extension from ultraviolet HfO₂ metasurfaces to visible-wavelength polymer metasurfaces therefore confirms that the GMT-based beam-shaping formulation remains applicable under substantially different refractive-index environments.

**S3. Sensitivity of the flat-top beam to the incident Gaussian divergence and robustness to other perturbations**

Within the combined geometric-transformation and IFTA-based design framework adopted in this work, the incident Gaussian beam is not treated as an external or arbitrary illumination condition, but constitutes an intrinsic boundary condition of the inverse design. Specifically, the target phase profile is explicitly derived from the cumulative energy distribution of a prescribed Gaussian beam, such that the transverse energy ordering of the input field is uniquely mapped onto that of the target flat-top beam under strict energy conservation. The corresponding one-dimensional geometric-transformation phase function is given by $\phi(x)=\frac{2\pi}{\lambda d}\int_{-D/2}^{x} \left[ \omega_{2}\left( \frac{S(x')}{S(D/2)}-\frac{1}{2} \right)-x' \right]dx';\left| x \right|<\frac{D}{2}$, where $S(x')=\int_{-D/2}^{x'} exp(-8\xi^{2}/\omega_{1}^{2})d\xi$ denotes the cumulative transverse energy distribution of the incident Gaussian beam. The normalized ratio $S(x')/S(D/2)$ therefore depends solely on the transverse intensity profile of the input field and establishes a one-to-one correspondence between the input Gaussian coordinate and the target flat-top coordinate.

The geometric mapping is constructed for a specific Gaussian beam characterized by a fixed waist position and half-divergence. When the incident Gaussian divergence is varied, the transverse intensity profile entering $S(x')$ is modified, thereby invalidating the originally designed energy correspondence. In addition, the Gaussian half-divergence $\theta$ determines the wavefront curvature of the incident field at the metasurface plane. Under the paraxial approximation, the divergence is related to the beam waist by $\theta\approx\lambda/(\pi\omega_{0})$, and the field contains an additional quadratic phase factor $exp\left[ {-ikx^{2}}/{(2R)} \right]$ associated with the wavefront curvature. Although this curvature term does not explicitly enter $S(x^{'})$, it modifies the local angular spectrum of the incident field and therefore affects the propagation consistency assumed in the geometric mapping. Because the phase function $\phi(x)$ is obtained through a double integration of the designed energy distribution, small deviations in the input divergence accumulate into significant changes in the curvature and slope of the phase profile. As a result, the originally designed one-to-one energy mapping is broken, leading to pronounced distortions of the flat-top beam, including plateau-amplitude variation, ripple formation, and effective beam-width change.

This sensitivity originates fundamentally from the geometric-transformation boundary condition and is further amplified by the nonlinear nature of the IFTA. In each iteration, the field is forward-propagated to the target plane, the target-plane amplitude constraint is imposed, and the phase is back-propagated to update the metasurface plane. The convergence of this high-dimensional, multi-parameter coupled process is strongly dependent on the assumed incident-field boundary condition. When the Gaussian divergence deviates from the design value, the initial field becomes inconsistent with the geometric-mapping-derived constraint, and the iterative process converges to a different local optimum, thereby amplifying the sensitivity of the final flat-top profile to the input beam divergence.

In contrast, variations in the incidence angle, illumination wavelength, and propagation distance act on different physical degrees of freedom and therefore have a comparatively weaker impact on the geometric energy mapping. A small incidence angle introduces an additional linear phase factor $exp(ikxsin\alpha)$ across the metasurface aperture, which primarily shifts the transverse momentum of the beam while preserving the monotonic transverse energy ordering encoded in $S(x')/S(D/2)$ . When implemented using Pancharatnam–Berry phase modulation, such linear phase perturbations can be tolerated within a moderate angular range without fundamentally altering the energy redistribution.

Variations in the illumination wavelength mainly rescale the propagation phase through $k={2\pi}/\lambda$ and the prefactor ${2\pi}/{(\lambda d)}$ in the phase function, leading to changes in efficiency and accumulated phase while leaving the spatial topology of the energy mapping largely unchanged. Similarly, modest deviations in propagation distance introduce a gradual quadratic phase mismatch through the Fresnel propagation kernel $exp\left[ {-ikx^{2}}/{2d} \right]$. Because the generated flat-top beam is designed to be quasi-collimated at the target plane rather than tightly focused, its transverse intensity distribution remains stable over a finite axial range around the design distance, and noticeable degradation occurs only when the accumulated phase mismatch becomes sufficiently large.

Overall, the pronounced sensitivity to the incident Gaussian divergence is an intrinsic consequence of the geometric-transformation-based inverse design, reflecting the high phase fidelity and precise energy mapping enforced by the metasurface. By contrast, the relative insensitivity to incidence angle, wavelength, and propagation distance arises from the fact that these parameters do not directly violate the transverse energy-ordering constraint embedded in the geometric mapping.
